# An affordable and optimized 3D biomodel for sinonasal surgery training

**DOI:** 10.1016/j.bjorl.2025.101750

**Published:** 2026-01-08

**Authors:** Marcelo Augusto Antonio, Sergio Lopes Fernandes Ramos, Fernando Augusto Lima Marson, Mariana Dalbo Contrera Toro, Eulalia Sakano

**Affiliations:** a Universidade Estadual de Campinas, Departamento de Oftalmologia e Otorrinolaringologia, Campinas, SP, Brazil; b Universidade Estadual de Campinas, Faculdade de Engenharia Mecânica, Campinas, SP, Brazil; c Universidade São Francisco, Laboratório de Microbiologia Clínica e Molecular, Laboratório de Biologia Molecular e Genética, LunGuardian Research Group ‒ Epidemiology of Respiratory and Infectious Diseases, Bragança Paulista, SP, Brazil

**Keywords:** 3D printing, Surgical simulation, Training model, Paranasal sinuses

## Abstract

**Objectives:**

To develop and evaluate the anatomical accuracy and haptic fidelity of an affordable kit consisting of a silicone head, a foam support, and a 3D-printed biomodel of the paranasal sinuses for surgical training.

**Methods:**

This single-center, technological, and interventional study detailed the development of a human sinus biomodel. Its anatomical and haptic evaluation was conducted by ten senior rhinologists, who performed 37 standard surgical tasks. Participants rated similarity to fresh cadaveric dissection on a 5-point Likert scale. Computed tomographic images were converted into a standard tessellation file through a combination of automatic and manual segmentation. This file was then used in a 3D printer to create the biomodel, which was customized with a red silicone coating to emulate mucosa, as well as silicone-insulated wire and acrylic yarn to simulate arteries and nerves. The model was mounted onto a silicone head and foam support, with a total production cost of US$ 43.37.

**Results:**

The similarity of anatomic landmarks scored a mean above 4.0 ± SD on both sides. The lacrimal bone drilling/debulking procedure was somewhat suboptimal for exposing the lacrimal sac, scoring a mean of 3.1 ± 1.04 on the left side but 4.3 ± 0.64 on the right. Likewise, uncinectomy was rated 3.4 ± 1.02 on the left and 3.7 ± 0.90 on the right, while the periorbit incision received scores of 3.5 ± 0.92 on the left and 4.5 ± 0.50 on the right. All other steps received ratings above 4.0 ± SD.

**Conclusion:**

This study presents a fully reproducible and cost-effective method for constructing a 3D sinus biomodel. The model demonstrated high anatomical and haptic fidelity, as validated by senior rhinologists, making it a valuable tool for surgical training in sinonasal procedures.

**Level of evidence:**

This study provides Level 2 evidence.

## Highlights

• An affordable set was developed for endonasal surgical training.
• Senior rhinologists rated its anatomical accuracy and haptics as realistic.
• The set offers a practical solution for repetitive surgical practice.

## Introduction

Studies have shown that hands-on training enhances surgeons’ ambidextrous dexterity, contributing to greater procedural safety and reduced surgery time.^1^^,^^2^ Achieving proficiency in surgical learning and minimizing complications involves significant lifelong costs. It is essential to consider not only material resources but also the time dedicated to foundational scientific studies and continuing education necessary to keep knowledge current.^2^

From their inception to the present day, medical schools have continuously sought effective methods to teach and improve surgical skills. While cadaveric training remains the gold standard, it presents several drawbacks, including ethical, religious, sanitary, and financial concerns. Alternative training methods include animal models, virtual reality simulators, and silicone or resin biomodels, which may or may not be produced using 3D printing technology. Although both virtual and physical models offer distinct advantages, their high costs and anatomical limitations remain significant challenges.^3^

3D printing has emerged as a promising alternative, enabling the creation of increasingly detailed anatomical replicas. Also known as additive manufacturing, this technique constructs objects layer by layer. The first method, stereolithography, was introduced in 1984 by Charles Hull, using ultraviolet light to polymerize resins.^4^ 3D-printed models are typically generated from computed tomography and magnetic resonance imaging scans using software such as Mimics 24.0 (Materialise; Leuven, Belgium) and OsiriX 14.1 (Pixmeo SARL; Geneva, Switzerland), which assist in converting medical image data into 3D-printable files. In otolaryngology, particularly rhinology, some 3D-printed models are designed to simulate internal carotid artery injury and bleeding control, allowing surgeons to refine their skills prior to real procedures.^5^^,^^6^

The most frequently used tool in rhinology and neurosurgery to assess the effectiveness of biomodels as simulators is the Likert scale, which evaluates aspects such as agreement, frequency, importance, similarity, probability, and quality. While biomodels benefit from technological advancements and often score highly on Likert scales, challenges remain regarding anatomical fidelity, tissue realism, and cost-effectiveness. In Brazil, where there are over 8,000 otolaryngologists and 298 first-year residents, the demand for accessible and high-quality training models is significant, underscoring the urgent need for continued innovation and investment in this area.^7^

Regardless of the standard operating protocol employed in biomodel creation, fundamental principles of health technology assessment ‒ such as efficacy, effectiveness, and efficiency ‒ should always be considered, along with continuous horizon scanning and technology monitoring. Fortunately, 3D printers are expected to become increasingly accessible, making biomodels more affordable and widely available.

Given the current scarcity and high cost of biomodels for Endoscopic Sinus Surgery (ESS) training ‒ such as those produced by Phacon (Leipzig, Germany)^8^ and Fusetec (Adelaide, South Australia)^9^ ‒ the authors aimed to develop and evaluate the anatomical accuracy and haptic realism of an affordable set for ESS. This set consists of a silicone head, a foam support, and a 3D-printed biomodel of the paranasal sinuses.

## Methods

A single-center study was proposed the Department of Ophtalmology and Otorhinolaryngology at the Faculty of Medical Sciences of the State University of Campinas (UNICAMP). All participants provided written informed consent. The study was approved by the University of Campinas Ethical Committee (CAAE: 53904921.0.0000.5404).

### Study design

This was a technological study aimed at creating a set consisting of a silicone head with a 3D biomodel of the paranasal sinuses and a head support, in addition to an interventional study involving evaluation by rhinologists.

### Standard operating protocol for biomodel development

The authors have filed a patent application with the National Institute of Industrial Property (nº BR 10 2025010414 8), and all the protocols detailing the required steps, materials, and procedures are available in the Supplement section to ensure the reproducibility of the set.

**Fig. 1:**
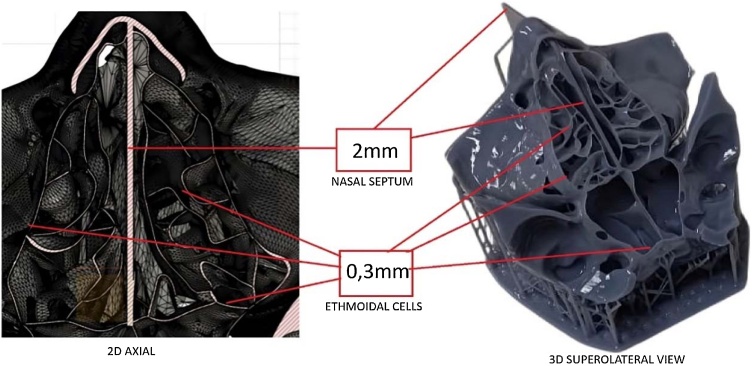
Two-dimensional and three-dimensional representations of the ethmoidal labyrinth based on computed tomographic data. Left: Axial computed tomographic highlighting the anterior and posterior ethmoidal cells. Right: superolateral view of the corresponding 3D-printed model in gray resin. All ethmoidal walls were printed with a uniform thickness of 0.3 mm, and a 2.0 mm nasal septum separates the two sides centrally.

Fig. 1 shows two-dimensional and three-dimensional representations of the ethmoidal labyrinth based on computed tomographic data used in the image modeling and 3D printing process.

### Peer evaluation by experts

The biomodel required hand-on manipulation for further validation. For this purpose, the study selected senior rhinologists from nine Brazilian universities: the State University of Londrina, the Federal University of Minas Gerais, the Federal University of São Paulo, the Federal University of Rio Grande do Sul, the Lutheran University of Brazil, the University of Brasília, the Santa Casa de São Paulo School of Medical Sciences, the State University of Rio de Janeiro, and the University of São Paulo (which contributed two participants).

Inclusion criteria required that evaluators be faculty members experienced in endoscopic nasal surgery and affiliated with a medical residency program. Exclusion criteria ruled out participants with known allergies to polyamide, rubber, or silicone, as well as those without access to a laboratory equipped for endoscopic nasal surgery simulation.

Each rhinologist received a package containing the set, along with a booklet titled *Standard Operating Procedure for 3D Biomodel Dissection.* The booklet outlined all the materials required for the dissection, followed by the sequence of tasks to be performed and evaluated using an answer sheet.

Initially, the rhinologist was informed: “Although there is no unique sequence for ESS, it is requested that each dissection step be followed in the stipulated order for methodological reasons. It is also advisable not to use surgical instruments other than those previously mentioned. Silicone cannot withstand cutting with sharp materials followed by traction; it tears easily. Rotations above 5.000 rotations per minute (r.p.m.) (or pressure above 4 Kgf/cm^2^ when using nitrogen), without irrigation, do not burn the silicone but can melt the 3D biomodel. The inferior turbinate will not be evaluated, nor will the nasal alae, which are only intended to support the endoscope”.

Once this was established, the rhinologist proceeded with tasks in the left nasal cavity, following the written instructions in the booklet: “Question #1: Start by positioning the head on the foam support as desired. Use the 30 ° endoscope. Check for any synechia between the middle turbinate and the nasal septum using the Cottle septum elevator or ball-seeker. Do not separate them yet. Gently medialize the middle turbinate using the Cottle septum elevator, avoiding the axilla. Check the uncinate process. Answer Question #1 and #2”.

Question #1 was designed to assess anatomic landmark accuracy, while Question #2 focused on the usefulness of the task for ESS training, as shown in Table 1. After each question, participants were asked to provide feedback using a 5-point Likert scale, ranging from 1 (strongly disagree) to 5 (strongly agree). This structure was applied to all 37 questions, which were grouped in Table 1 according to their focus on either anatomical accuracy or task relevance for surgical training.

**Table 1:** Answers among evaluators regarding anatomical accuracy and biomodel’s usefulness.

Question	Anatomic accuracy	Number of answers on likert scale					Mean rating score (SD)
		1	2	3	4	5	
1	Left paradoxical middle turbinate and uncinate process				4	6	4.6 (0.49)
3	Left ethmoidal bulla and ethmoidal infundibulum space				4	6	4.6 (0.49)
7	Left basal lamella			1	6	3	4.2 (0.60)
10	Left sphenoid sinus, carotic artery bulge, and optic nerve canal				5	5	4.5 (0.50)
19	Left frontal sinus		1		5	4	4.2 (0.87)
20	Right concha bullosa and the prominent *agger nasi*				6	4	4.4 (0.49)
22	Right uncinate process and ethmoidal infundibulum				5	5	4.5 (0.50)
34	Final aspect of Draf III		1	1	3	5	4.2 (0.98)
	Usefulness of the task						
2	Left middle turbinate medialization			1	6	3	4.2 (0.60)
4	Left uncinectomy		2	1	5	1	3.4 (1.02)
5	Left maxillary antrostomy		1	2	4	3	3.9 (0.94)
6	Left bullectomy and anterior ethmoidectomy			1	6	3	4.2 (0.60)
8	Left posterior ethmoidectomy				9	1	4.1 (0.30)
9	Left transethmoidal sphenoidotomy			1	6	3	4.2 (0.60)
11	Left squeletization and fracture of the lamina papyracea			1	4	5	4.4 (0.66)
12	Left periorbit incision		2	2	5	1	3.5 (0.92)
13	Left lacrimal bone drilling/debulking for lacrimal sac exposure	1	2	2	5		3.1 (1.04)
14	Left use of a silver pin to mark the location of the lacrimal sac and common canaliculus	2		5	3		2.9 (1.04)
15	Left Draf I				8	2	4.2 (0.40)
16	Left Draf IIA			1	7	2	4.1 (0.54)
17	Left Draf IIB			1	6	3	4.2 (0.60)
18	Left: use of an acrylic yarn to identify the first olfactory fibers		1	3	3	3	3.8 (0.98)
21	Right concha bullosa reduction			2	4	4	4.2 (0.75)
23	Right uncinectomy		1	3	4	2	3.7 (0.90)
24	Right maxillary antrostomy		1	1	3	5	4.2 (0.98)
25	Right anterior ethmoidectomy			1	5	4	4.3 (0.64)
26	Right posterior ethmoidectomy			1	6	3	4.2 (0.60)
27	Right transethmoidal sphenoidotomy				5	5	4.5 (0.50)
28	Right excision of part of the lamina papyracea and periorbit incision				5	5	4.5 (0.50)
29	Right lacrimal bone drilling/debulking for lacrimal sac exposure			1	5	4	4.3 (0.64)
30	Right Draf I		1	2	4	3	3.9 (0.94)
31	Right Draf IIA				5	3	4.5 (0.50)
32	Right Draf IIB				3	7	4.7 (0.46)
33	Draf III				2	8	4.8 (0.40)
	Availability of biomodels and dexterity training						
35	Lack of biomodels for Endoscopic Sinus Surgery (ESS) training in Brazil		1	1	5	3	4.0 (0.89)
36	Lack of affordable biomodels for ESS training in Brazil				3	7	4.7 (0.46)
37	Bimanual dexterity is acquired through repetitive training rather than sporadic exposition				2	8	4.8 (0.40)

For each of the 37 basic items related to Endoscopic Sinus Surgery (ESS) training, note the number of times a score was assigned based on a 5-point Likert scale (columns from 1 to 5), along with the mean value and Standard Deviation (SD) of the responses.

The final three questions assessed agreement with the following statements: “There is a lack of biomodels for ESS training in Brazil (affordable or not)” and “Bimanual dexterity is acquired through repetitive training rather than sporadic exposure”. At the end of the answer sheet, participants were also invited to respond to an open-ended question: “Comment on how you could improve this biomodel” (Table 1). Responses were submitted anonymously via the phone number provided in the informed consent form.

### Statistical analysis

Responses were compiled using Excel® (Microsoft Excel; Microsoft Corp., Redmond, WA, USA). The statistical analysis was performed using SPSS (Statistical Package for the Social Sciences) software (IBM SPSS Statistics, version 28; IBM Corp., Armonk, NY, USA). The mean and standard deviation were calculated for each response.

## Results

A total of 10 rhinologists were included in the analysis, as they returned fully completed answer sheets. One rhinologist omitted a single response, which was subsequently considered neutral and assigned a score of “3”. Table 1 shows how many times each response option on the Likert scale was selected for each question.

Regarding anatomical accuracy of the bullous conchae, uncinate process, maxillary and sphenoid antrum, frontal recess, and the final aspect of the Draf III procedure, none of the 37-items described in Table 1 had a mean score below 4.0 ± SD on either sides.

Concerning the criterion of task usefulness, only one item received a mean score below 3 (neutral): 2.9 ± 1.04, which referred to the use of a silver pin to mark the location of the lacrimal sac and common canaliculus. Similarly, the rhinologists found the lacrimal bone drilling/debulking procedure suboptimal for exposing the lacrimal sac, with a mean score of 3.1 ± 1.04 on the left side. Uncinectomy was rated 3.4 ± 1.02 on the left and 3.7 ± 0.9 on the right, while the periorbit incision received scores of 3.5 ± 0.92 on the left and 4.5 ± 0.5 on the right. All other steps, whether performed with manual or powered instruments, scored above 4.0 ± SD.

There was consensus that bimanual dexterity is developed through repetitive training rather than sporadic exposure, as reflected by a mean score of 4.8 ± 0.4. Regarding the statement about the lack of biomodels for ESS training in Brazil, the initial mean score was 4.0 ± 0.89, which increased to 4.7 ± 0.46 after the word “affordable” was added to the statement.

In response to the open-ended question, “Comment on how you could improve this biomodel”, 9 out of 10 participants suggested improving the consistency of the mucosa, while 7 out of 10 recommended enhancing the elasticity of the nasal tip by using a more malleable silicone.

The costs for producing one complete set are detailed in Table 2.

**Table 2:** Quantity and cost of supplies required to produce one head-biomodel-support set.

Supply	Price (US$)^a^	Units per set	Price per set (US$)^a^
3D printer	800.00 (unit)	1	Permanent facility
White acrylate resin	43.5 per L	50 mL	2.17
70% alcohol	1.74 per L	1 L	1.74
Cristal acetic silicone	2.45 per 50g	50g	2.45
Liquid silicone	5.5 per 300g	20g	0.36
Crystal platinum silicone	52.24 per Kg	0.07 Kg	3.65
White silicone rubber	12,19 per Kg	2.7 Kg	26.17
Beige silicone dye	5.22 per 100g	15g	0.78
Red silicone dye	3.87 per 100g	2g	0,07
Foam support	2.26	1	2.26
Stationery for Standard Operating Procedure	3.48	1	3.48
Customization (materials)	24.93/100 pieces	1	0.24
			**Total gross US$ 43.37**

All materials used for production, including customization items, are detailed. Customization with foam fragments, acrylic yarn, silicone-insulated wire, pins, and rayon venules was estimated at US$ 0.24 per biomodel, based on the production of 100-units. For this quantity, the following materials were used: 200-pins (US$ 2.61), 10 m of yellow acrylic yarn (US$ 1.95), 8 m of red silicone-insulated wire (US$ 2.02), 6 m of yellow silicone-insulated wire (US$ 1.53), 200 round foam pieces (US$ 5.85), and 4 g of rayon filaments (US$ 10.45), totaling US$ 24.42.

a Currency conversion date March 15, 2025: 1 US$ = R$ 5,74.

## Discussion

The Likert scale responses in this study were based on comparisons each rhinologist made with their prior experiences using cadavers and other biomodels. A 5-point Likert scale was chosen over a 9-point version to simplify the decision-making process between opposing viewpoints, making it easier to interpret as approval or disapproval of the set. Upon approval, the set could be mass-produced for broader evaluation, assessing its impact on residents’ surgical training.

**Fig. 2:**
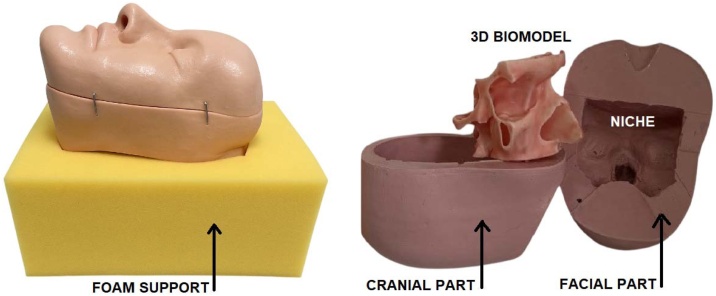
Three-piece assembly of the training set. On the left, the silicone head is secured by the foam support. On the right, the two silicone head segments and the central niche designed to accommodate the 3D-printed biomodel are shown.

Overall, since most scores exceeded 4.0 ± SD on a scale ranging from 1 (strongly disagree) to 5 (strongly agree), the evaluation supports the biomodel’s anatomical accuracy and its suitability for ethmoidal and frontal sinus manipulation. As in previous studies,^10^, ^11^, ^12^, ^13^, ^14^ this model was based on tomography images. However, extensive digital sculpting was pivotal to achieving very thin, yet bone-like rigid, ethmoidal cells (Fig. 2). Reproducing the haptics of the ethmoidal labyrinth proved to be a trial-and-error process involving the testing of multiple wall thicknesses and different resins. This iterative approach required close collaboration between a Blender 3D (Blender Foundation; Amsterdam, Netherlands) modeler and the anatomical expertise of the authors, ultimately extending the development time. Each pilot model required further refinements after printing.

**Fig. 3:**
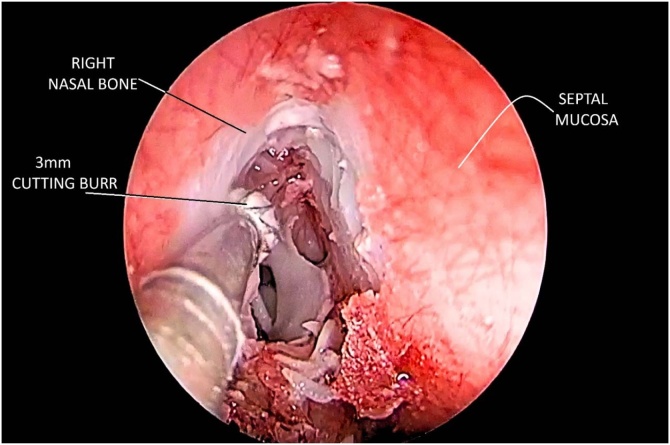
Endoscopic view during the simulation of drilling access to the frontal sinus. The whitish area simulates exposed bone after detaching the red mucosal layer. A 3 mm cutting burr is drilling out the frontal beak region. Fragments of synthetic bone and mucosa are visible at the bottom.

Due to its low cost and ease of acquisition, acrylic resin was an accessible material for testing and demonstrated high applicability, even for very thin walls. It provided an ideal balance of hardness and flexibility for both manual and powered instruments, without melting or producing powder that could blur the lens, as observed with other materials like polyamide.^12^ However, no acrylic resin can withstand high-speed drilling indefinitely without beginning to melt. This limitation is not always disclosed by biomodel suppliers. In our study, drill speeds were limited to 5,000 rpm (or drilling pressure of up to 4 Kgf/cm^2^ using nitrogen) and required irrigation to prevent melting. (Fig. 3) (Video supplement).

Mucosal haptics, especially during uncinectomy and manipulation of the lacrimal bone, lacrimal sac, and periorbita incision, received the lowest scores among the 37 evaluated items, indicating limited utility for surgical training. Achieving realistic width and flexibility for mucosa and the lacrimal sac/duct remains a challenge. Multi-material printers offer potential by layering materials with different textures to simulate bone and mucosa.^15^ However, such printers are still expensive and not widely accessible. The authors tested materials such as VeroWhitePlus/TangoPlus (Stratasys; Eden Prairie, USA), as used in a previous study.^15^ Although the hardness of the bony components was partially satisfactory, several ethmoidal cells were overly rigid and had their lumens inadvertently filled with resin.

There are studies focused on 3D biomodels designed primarily for drilling, often without a mucosa layer.^5^^,^^11^^,^^12^^,^^16^ In contrast, our study tested different materials to simulate mucosa, including ultracentrifuged latex, platinum silicone, thermoexpandable paint, latex paint, and liquid rubber, either alone or in various combinations. We noted that some of these materials could not cure, be detached, or fill thin spaces, and even merged with the resin of the bony parts, compromising final hardness. However, the acetic silicone used for this biomodel did not compromise such aspects.

Although new simulators are often considered useful by residents and/or senior rhinologists, their limited availability likely stems from high production costs or lack of investment in large-scale manufacturing.^5^^,^^6^^,^^11^^,^^17^^,^^18^ The high price of biomodels limits opportunities for repeated practice. In this study, rhinologists agreed that repetition is essential for effective surgical training. From its inception, a key objective in developing this biomodel was to avoid techniques and materials that would unnecessarily increase costs. For example, the foam support needed to accommodate various head positions, contain fluids when necessary, and remain lightweight to reduce shipping costs. Many simulators require secure fixation to specific supports.^8^^,^^9^^,^^12^ Furthermore, materials were selected with long-term durability in mind, ensuring the model’s relevance amid technological advancements ‒ an important consideration before investing in more expensive solutions, such as augmented or virtual reality.^3^

Although the total production cost was significantly reduced, manual labor for head and support assembly and post-processing was not included, as it can vary depending on commercial scale. The head required artisanal work, but once its molds were finalized, mass production became feasible. Despite being the most expensive component, the 3D printer used was one of the simplest models, costing approximately US$ 800.00.

The relatively small number of evaluators is a limitation of the study. Additionally, relying solely on experienced specialists rather than including undergraduates or residents introduces potential bias. Nevertheless, their participation offered the advantage of drawing upon substantial experience with live surgeries, cadaver dissections, and widely used commercial biomodels. The absence of simulated blood and cerebrospinal fluid also represents a limitation.

It is worth noting that anatomical features or variations, such as tumor lesions or bone dehiscence/thickening, could be added to the file using Blender 3D, thereby enhancing its versatility for surgical simulation.

## Conclusion

It is feasible to produce cost-effective 3D biomodels that incorporate anatomical variations, enhancing their value as educational tools, particularly for developing the psychomotor skills of Otolaryngology residents and contributing to the shortening of surgical learning curves.

## ORCID ID

Marcelo Augusto Antonio: 0000-0002-5858-1229

Sergio Lopes Fernandes Ramos: 0009-0001-6931-453X

Fernando Augusto Lima Marson: 0000-0003-4955-4234

Mariana Dalbo Contrera Toro: 0000-0002-5294-369

Eulalia Sakano: 0000-0002-5963-912X

## Funding

The research did not receive any specific grant from funding agencies in the public, commercial, or not-for-profit sectors. All expenses were covered by the first author, including the production of the video described in the Methods section, silicone heads, foam supports, dissection manuals, shipping to evaluators, as well as all the silicone used in the unsuccessful attempts to create the silicone head.

## Data availability statement

We declare that all data is available in the repository.

## Declaration of competing interest

The authors declare no conflicts of interest.
