# Supplementary material for: An affordable and optimized 3D biomodel for sinonasal surgery training
Source: Braz J Otorhinolaryngol. 2026 Jan 8;92(2):101750. doi: 10.1016/j.bjorl.2025.101750 (PMC12818127; doi:10.1016/j.bjorl.2025.101750)
Supplement: Supplementary file 1 [file mmc1.docx]

**BJORL-D-25-00220**

**Material Supplementary**

**STANDARD OPERATING PROTOCOL FOR BIOMODEL DEVELOPMENT**

1. **Acquisition of Imaging Data**: Anonymized computed tomography of human nasal cavities and paranasal sinuses were obtained using a 128-slice computed tomography Scanner (Philips Brilliance; Best, Netherlands). Scanning was performed with a slice thickness of 0.5 mm and a slice interval of 0.1 mm, resulting in an overlap of 0.4 mm to ensure high-resolution imaging suitable for 3D reconstruction.
2. **Image Conversion and Initial Processing:** Computed tomography images were converted into DICOM format (Digital Imaging and Communications in Medicine, NEMA PS3/ISO 12052; Rosslyn, USA) for compatibility with medical imaging software. The files were processed using InVesalius 3.1.1 (.inv file) (Centro de Tecnologia da Informação; Campinas, Brazil) and Mimics 24.0 (.mcs file) (Materialise; Leuven, Belgium) to enable automatic segmentation of anatomical structures including bone, cartilage, soft tissues, and air-filled cavities.
3. **Manual Segmentation:** Following automatic segmentation, each image slice was manually reviewed in the coronal, axial, and sagittal planes. The corresponding .inv and .mcs files were verified to ensure accurate anatomical representation, with particular focus on the intricate segmentation of the ethmoid labyrinth **(Figure 1).**
4. **3D Surface File Generation:** Mimics software generated a 3D surface in .stl format. This file was then prepared for 3D printing and uploaded to an ANYCUBIC Mono 2 printer (Anycubic SLA-LCD 4K; Shenzhen, China), which uses stereolithography for high-resolution anatomical modeling.
5. **Digital Modeling and Refinement:** Further digital modeling was conducted using Blender 3.6 (Blender Foundation; Amsterdam, Netherlands). This step involved reconstructing the nasal septum, the walls of the ethmoid and sphenoid sinuses, the lamina papyracea, and the maxillary bones. Additionally, resin drainage holes were created to optimize the printing process and post-processing workflow.
6. **Adjustment of 3D Printing Parameters:** The 3D printing process for each biomodel was optimized with an average print time of approximately 5 hours. The following parameters were used:
7. **Material:** White acrylic monomer resin (405 nm wavelength) (Anycubic; Shenzhen, China).
8. **Polymerization**: Achieved using ultraviolet light at 405 nm.
9. **Exposure Time per Layer**: 3.5 seconds.
10. **Layer Thickness:** 0.03 mm.
11. **Printing Orientation**: 45° angle relative to the X-axis. It enhanced structural integrity and minimized support artifacts.
12. **Post-Processing Procedure After Printing**:  approximately 30 minutes per biomodel
13. Immersion in 70% alcohol for 20 minutes to remove uncured resin residues.
14. Drying at 28°C for 10 minutes to ensure complete evaporation of alcohol.
15. Manual removal of supports using precision tweezers.
16. No additional ultraviolet light curing was necessary.
17. **Room Temperature Drying:** The biomodels were allowed to dry at a dark, ambient room temperature to ensure complete stabilization before further handling
18. **Simulation of Olfactory Nerve:** A yellow acrylic yarn (Anne 65; Santa Catarina, Brazil) was positioned in the anterior region of the cribriform plate to simulate the first branch of the olfactory nerve. The yarn was fixed in place using a coat of cyanoacrylate adhesive applied to the superior surface of the cribriform plate.
19. **Simulation of Neurovascular Structures**
20. A 3 mm yellow silicone-insulated wire (Caboflex; Marília, Brazil) was inserted into the optic nerve canal to represent the optic nerve.
21. A 4 mm red silicone-insulated wire was positioned to simulate the cavernous segment of the internal carotid artery.
22. **Simulation of Lacrimal System Probing:** A silver pin was inserted into the lumen of the lacrimal sac and duct to simulate probing, allowing tactile feedback for surgical training.
23. **Preparation of Colored Silicone Coat**: Preparation of transparent acetic silicone (Tekbond, Embu das Artes, Brazil) mixed with liquid silicone (Tekbond, Embu das Artes, Brazil) and red silicone dye (CAS 6829-22-7) (Redelease, Barueri, Brazil) in a ratio of 10:4:0.2, respectively; rayon filaments (Clássico, Campo Limpo Paulista, Brazil) were added to emulate veins in an amount that preserved the fluidity of the mixture. This composition was apllied to all internal regions of the biomodel: three coats on the nasal septum, floor, olfactory fossae, and maxillary lines, and two coats on the remaining areas of the nasal fossae.
24. **Simulation of the Periorbit:** Two coats of the previous mixture were applied over the external portion of the lamina papyracea to simulate the periorbit.
25. **Simulation of the Nasal Bone Periosteum:**  Three coats of the mixture were applied, with extra red dye (10:0,4).
26. **Handcrafted Production of the Craniofacial Model:** The craniofacial model was handcrafted in two separate parts (the cranial and facial portions) using beige liquid silicone rubber (Redelease; Barueri, Brazil). Prior to pouring the silicone into the facial mold, the nasal region was pre-filled with flexible platinum silicone rubber, also tinted with beige silicone dye at a ratio of 10:0.5, to enhance more flexibility in that area.
27. **Simulation of Periorbital Fat:** The orbital cavities were filled with spherical foam fragments (density: 13 kg/m³) to simulate periorbital fat.
28. **Placement into Facial Mold**: The 3D biomodel was inserted into the facial portion of the silicone head mold (**Figure 2**).
29. **Head Assembly:** The cranial and facial portions of the head were joined using a male-female fitting system to ensure structural stability (**Figure 2**). .
30. **Surgical Positioning and Foam Support**: For surgical positioning, the assembled head was mounted on a custom foam support (density: 33 kg/m³). Two foam pieces were bonded with contact adhesive (Loctite, São Paulo, Brazil) to form a waterproof internal niche, preventing fluid leakage during wet procedures (**Figure 2**).
31. **Final Anatomical Verification**: A final computed tomography scan (as per Step 1) was performed on the complete biomodel to confirm anatomical accuracy. The resulting images were emailed to the rhinologists for review.
32. **Replication of the Set**: Ten identical replicas of the full training set were produced by repeating Steps six through 19.
